# Oncogenes and cancer associated thrombosis: what can we learn from single cell genomics about risks and mechanisms?

**DOI:** 10.3389/fmed.2023.1252417

**Published:** 2023-12-20

**Authors:** Nadim Tawil, Abdulshakour Mohammadnia, Janusz Rak

**Affiliations:** ^1^Research Institute of the McGill University Health Centre, Montreal, QC, Canada; ^2^Neuroimmunology Unit, Montreal Neurological Institute, Department of Neurology and Neurosurgery, McGill University, Rue University, Montreal, QC, Canada

**Keywords:** cancer-associated thrombosis, thrombosis, cancer, venous thromboembolism, oncogenes, coagulome, single-cell sequencing, multi-omics

## Abstract

Single cell analysis of cancer cell transcriptome may shed a completely new light on cancer-associated thrombosis (CAT). CAT causes morbid, and sometimes lethal complications in certain human cancers known to be associated with high risk of venous thromboembolism (VTE), pulmonary embolism (PE) or arterial thromboembolism (ATE), all of which worsen patients’ prognosis. How active cancers drive these processes has long evaded scrutiny. While “unspecific” microenvironmental effects and consequences of patient care (e.g., chemotherapy) have been implicated in pathogenesis of CAT, it has also been suggested that oncogenic pathways driven by either genetic (mutations), or epigenetic (methylation) events may influence the coagulant phenotype of cancer cells and stroma, and thereby modulate the VTE/PE risk. Consequently, the spectrum of driver events and their downstream effector mechanisms may, to some extent, explain the heterogeneity of CAT manifestations between cancer types, molecular subtypes, and individual cases, with thrombosis-promoting, or -protective mutations. Understanding this molecular causation is important if rationally designed countermeasures were to be deployed to mitigate the clinical impact of CAT in individual cancer patients. In this regard, multi-omic analysis of human cancers, especially at a single cell level, has brought a new meaning to concepts of cellular heterogeneity, plasticity, and multicellular complexity of the tumour microenvironment, with profound and still relatively unexplored implications for the pathogenesis of CAT. Indeed, cancers may contain molecularly distinct cellular subpopulations, or dynamic epigenetic states associated with different profiles of coagulant activity. In this article we discuss some of the relevant lessons from the single cell “omics” and how they could unlock new potential mechanisms through which cancer driving oncogenic lesions may modulate CAT, with possible consequences for patient stratification, care, and outcomes.

## Introduction

It is becoming increasingly clear that even clinically localized (non-metastatic) cancers exert widespread systemic influences upon the function of multiple organs and tissue compartments (1), including the hemostatic system (2). These influences may vary from subtle laboratory changes to clinically manifest morbid conditions, such as VTE, ATE, PE, disseminated intravascular coagulation (DIC), or bleeding conditions, which can be exacerbated by therapy, metastatic progression and other factors usually worsening patients’ outcomes and, in some cases, contributing to cancer-related mortality (2). The clinical, hematological, and pathophysiological underpinnings of the related procoagulant states, often referred to, collectively, as cancer-associated thrombosis (CAT), have received extensive analytical attention, as did traditional diagnostic approaches, strategies of implementing thromboprophylaxis and treatment used in medical practice, or in clinical trials (2). In this commentary we will not cover these well studied aspects of CAT, but instead we will focus on questions surrounding the upstream triggering mechanisms by which cancer cells and their complex populations may project their influence and dynamic diversity upon the hemostatic apparatus leading to its perturbations in cancer patients. While this discussion is not intended to present original data, it delves into the treasure trove of information contained in published molecular profiling datasets, which we occasionally mine, to illustrate the points of particular interest. The conceptual axis of this piece is the notion that cancer-causing mutations and transforming epigenetic alterations are ultimately implicitly involved in CAT and we explore how the increasing understanding of oncogenic processes may, by extension, illuminate some of the underappreciated and potentially actionable aspects of cancer-related prothrombotic states.

## The quest to understand cancer associated thrombosis

Armand Trousseau (1801–1867), wrote with fascination about the frequency at which his patients with malignancy were also afflicted with a painful oedema in their upper or lower extremities (3). While certainly remarkable, his observation did not explain the nature or consequences of this intriguing ailment. Trousseau was initially perplexed as to the cause-effect relationship between these two conditions. He noted that in many cases patients presented with painful oedema first, and only upon autopsy, a cancerous mass was discovered, often at visceral locations, without previously manifested symptoms (3). Trousseau syndrome, a term that signifies abnormal blood clotting linked to an underlying occult malignancy (4) ultimately became a famous eponym (5). The term CAT represents the link between cancer and thrombosis more broadly regardless of the sequence that led to diagnosis of either. Indeed, over the years this linkage was recognized for its multiple dimensions including tumor microvascular thrombosis, as well as macrovascular, that is, arterial (ATE) or venous thromboembolism (VTE), the latter being most studied (2).

Implicitly, CAT in all its systemic forms (VTE, ATE, and PE) must involve a communication between cancer-related triggering process and target vascular sites in which thrombosis may occur. While this is an area of an ongoing debate, factors that may mediate such long range interactions may include coagulant fragments of cancer or stromal cells such as extracellular vesicles (EVs) or particles (EPs), and their fractions traditionally referred to as microparticles (MPs) from various cellular sources (6–9). The suggested relevant cargo of EVs/EPs may include tissue factor (F3/TF), podoplanin (PDPN), and other canonical hemostatic molecules or entities of less studied nature, such as polyphosphate, mucins, genomic DNA, and other potentially coagulant classes of molecules. The systemic effects of CAT could also involve changes in circulating myeloid cells, platelets, and plasma hypercoagulability for which structural features of peripheral blood vessels may constitute triggers for the onset of thrombosis (2, 10). It is therefore of some relevance not only what coagulant molecules cancer, or host cells express themselves during the disease process, but also what are the molecular mechanisms triggered by cancer and still largely unknown, by which the effects of these CAT-driving factors may be systemically disseminated. Both would likely contribute to thrombosis risk causation and prediction.

The association between cancer an VTE has been solidified over the years including in patient cohorts of multiple different cancer types, or with specific cancers (2, 11–15). For example, in a study comprising 21,002 new cases of VTE spanning the period between 1995–1996, as many as 20.78% of the VTE cases were associated with malignancy (16, 17). Another report based on a more recent large patient registry, the Registro Informatizado de Enfermedad Trombo Embólica (RIETE) including over 35,000 VTE cases diagnosed between 2001 and 2011, pinpointed the presence of cancer in 17% of cases (18). A more general overview of data from various sources suggests that in 15–25% of VTE cases, a malignant disease is ultimately found (2, 19–22).

Conversely, cancer diagnosis may increase the risk of VTE. In this regard, recent work based on a Danish cohort of 499,092 patients with first time cancer diagnosis spanning the years between 1997 and 2017 has reported an elevated VTE incidence, particularly throughout the span of 6 months prior to, and following, cancer diagnosis. In this case the risk of VTE was up to 12-fold higher in cancer patients compared to the general population (23). A troubling observation is the seemingly increasing incidence of cancer associated VTE despite improved cancer therapy outcomes (23–27). In 2021, the population-based cohort study by Mulder et al. (23) highlighted that among cancer patients, those receiving chemo- or targeted therapy, exhibited nearly double the risk of VTE, which was up to 23-fold higher when compared to the general population. Equally, a striking increase in VTE was being registered by studies conducted over the past 20 years; whereby, the 12 months cumulative VTE incidence rose three-fold among cancer patients overall, and six-fold among recipients of chemo- or targeted therapy (23). This is important because VTE remains one of the leading causes of death among certain cancer patients (28, 29) and recent autopsy data involving 9,571 cancer patients highlighted a high prevalence of pulmonary embolism among the deceased (PE; 12.4%). PE was identified as a contributing factor to death in 29.3% of patients at autopsy, and as a primary cause of death in another 37.7% of patients in clinical care (2, 30). Thus, CAT brings about a considerable burden of disease and poor outcomes, along with the necessity to monitor and treat patients. CAT management and treatment modalities are currently not predicated on the nature of the underlying malignancy, but instead stem from largely hematological considerations, a reality that may be worth reconsidering.

## Diverse manifestations of CAT within the landscape of human malignancies

The overall risk of cancer-associated VTE varies across different studies, patient cohorts and tumor types. Elevated VTE risk could be a consequence of a number of factors such as age, ethnicity, underlying conditions, metabolic considerations, therapeutic modalities, and proportions of different cancer types within the respective cohorts (31). The perceived contributions of these factors to CAT may also be affected by study design, which involves statistical approaches, inclusion/exclusion criteria and definitions of end-points (15).

Although cancer has been conclusively linked to higher overall risk of VTE, different cancer types exhibit different propensities to promote a procoagulant state (15, 32–35). Indeed various manifestations of malignancy bring on different levels of predisposition for thrombosis, ranging from a high VTE risk cancers (pancreatic, glioblastoma, ovarian and hematopoietic) to moderate (lung, high grade melanoma, and bladder) to low VTE risk malignancies (prostate, renal and breast) (10, 11, 36). Such an uneven impact suggests that the inherent properties and cellular, molecular and genetic composition within different cancers could have a direct or indirect influence on the development and pathogenesis of CAT (35).

### Oncogenes and VTE effectors

Potential molecular mediators of CAT, now often referred to as “cancer coagulome” (37), have been found to exhibit stark differentials in their expression patterns across different cancer types (38). While at a first glance such differences could be attributed to stochasticity and/or organ-specificity, as well as particularities of tumor microenvironments, the non-random differentials in VTE risks and coagulome composition across molecular/histological subtypes and grades of a particular cancer has been repeatedly observed and reported (33).

In this context, brain tumors, such as gliomas may represent an informative example of the link between CAT and salient features of cellular transformation (39–45). Notably, a staggering variance in prothrombotic impact exists in glioma as a function of disease grade (e.g., low-grade vs. high-grade disease) (41, 46). High-grade glioma (HGG), particularly grade IV that includes glioblastoma (GBM), is the most aggressive form of primary brain tumors, with GBM being associated with one of the highest risks of VTE (20%–30%) systemically (41, 47). Moreover, GBMs manifest a consistent (and diagnostic) presence (~90%) of intratumoral microthrombosis (48–52). This is contrasted by a VTE risk of 8.2% for grade II and 9.2% for grade III gliomas (41) with correspondingly lower frequency of microthrombosis (52). Thus, both heightened VTE risk and extent of intratumoral microthrombosis are associated with HGG, albeit with qualifications dependent on the molecular landscape of the disease (35).

Within the HGG diagnosis the extent of CAT, including the VTE risk is strongly influenced by molecular drivers of the disease. While this topic is extensively reviewed elsewhere (35, 53), it is worthwhile reciting some of the key aspects in brief. In this regard one of the major determinants of the VTE risk in HGG is the presence or absence of isocitrate dehydrogenase 1 or 2 (IDH1/2) mutations, with wild type IDH1/2 status identifying procoagulant glioblastomas (GBMs) and IDH1/2 (R132H) mutation associated with a relatively non-coagulant presentation (47). The IDH1 wild type HGG tumours presently referred to as proper GBM, are further subclassified into classical (CL), mesenchymal (MES) and proneural (PN) subgroups in part depending on major genetic drivers of oncogenesis, particularly EGFR (CL), NF1 (MES), and PDGFRA (PN), as well as transcriptomic signatures and methylation patterns (54).

Notably, the aforementioned GBM subtypes are associated with distinctive repertoires of coagulation-related genes (39). For example, PN-, MES- and CL-type tumours markedly differ in the expression of F3/TF (tissue factor) gene encoding the central cell-associated, transmembrane receptor and regulator of the extrinsic coagulation cascade (55). TF acts as the main activator of the circulating coagulation factor VII (FVII) and the resulting complex triggers formation of active coagulation factor Xa (FXa), and thrombin (FIIa), the latter of which drives polymerization of plasma fibrin monomers, the main substrate in the blood clotting process and a key constituent of intravascular thrombi. Thrombin also potently activates platelets, which also accumulate in clots, and it interacts with cellular signalling receptors (e.g., protease activated receptor 1-PAR-1) on various cells whereby it exerts multiple regulatory effects across the tumour microenvironment (56). Platelets are also activated by podoplanin (PDPN), another membrane protein present on the surface of various cells including GBM cells (6, 57). Procoagulant effects in cancer may also be induced by the expression of plasminogen activator inhibitor 1 (PAI-1), and by recruitment of inflammatory cells through cytokine release (10, 58). Several of these proteins (TF, PDPN, PAR1, FVII, and other effectors) of the hemostatic system are expressed by GBM cells, often ectopically and in a manner that impacts the cell-associated procoagulant activity, and is correlated with their molecular subtype and oncogenic programs (33, 59).

Because GBMs are heterogeneous with respect to their molecular make up and oncogenic wiring it could be reasoned that the globally increased VTE risk and microthrombosis could be triggered by diverse effector mechanisms. Such mechanisms could involve various aforementioned molecular mediators, of which TF and PDPN are among the most studied, but not the only candidates (6). While both TF and PDPN are targets of epigenetic and genetic oncogenic regulation (6, 47, 60) they may act in concert with each other and with other mediators expressed by altered cancer cells, or by the “activated” tumour stroma, and at the systemic level due to paracrine influence (61), thus potentially adding to the disease complexity. For example, cancer-driven leukocytosis, and neutrophilia may result in formation of procoagulant, chromatin-containing neutrophile extracellular traps (NET), which may contribute to CAT in various disease settings (10, 35).

While the multiplicity of changes associated with CAT may create a sense of great molecular complexity, or stochasticity, it should be noted that properties of the tumor microenvironment are, to some extent, defined by the consequences of the oncogenic transformation that triggers and propels cancer cell growth, invasion, and, importantly, also interactions with other cellular populations, as well as the composition of the cellular transcriptome, secretome and coagulome. In fact, experimental studies demonstrate that changes in the status of a single mutant oncogene may trigger complex changes in the coagulation apparatus of affected cells (59, 62). Along these lines the influence of cancer associated genetic alterations on the promotion of a tumor-induced thrombogenic state and thus risk of VTE is being actively studied and frequently reviewed (35, 36, 53, 63). A number of studies also examined the link between specific mutant oncogenic drivers and VTE risk. In this regard the most widely explored associations include the alterations in ALK (44, 64, 65), KRAS (66–68), and ROS1 (69–71) among other oncogenes (36).

### VTE beyond oncogenic mutations

While oncogenic drivers have been implicated in triggering a procoagulant phenotype in various cancers, their presence is not a sole, strong, or universal predictor of the VTE risk in a given cancer. For instance, KRAS has been implicated in driving an aberrant overexpression of TF, as well as in promoting TF release as active cargo of cancer-derived extracellular vesicles (EVs) (62, 72–74). In keeping with this observation KRAS mutations have been found by several studies to be associated with increased VTE risk across a spectrum of human malignancies, namely in colorectal (36, 66), lung (36, 67, 75), and pancreatic cancers (36, 76). However, it is worth noting that some other studies examining colorectal and lung cancers failed to observe this correlation (63, 75, 77–81). In pancreatic cancer, reporting on KRAS, as a direct correlate of VTE is relatively scarce, despite both being generally prevalent in this disease setting (63, 68, 82). This is informative since mutations of KRAS are found in the vast majority of exocrine pancreatic ductal adenocarcinomas (PDACs) without inevitable occurrence of VTE (11). In the other words, in this setting KRAS mutations are reported with a prevalence range of 90%–95% (83), yet VTE incidence, which is often gauged by overt clinical presentation, reaches a much lower level of 20%–40% (11, 17, 84–86). Therefore, it could be argued that the procoagulant impact of oncogenic KRAS may either be subclinical in nature or, could be counteracted by yet unidentified factors. It is also possible that the effects of KRAS may be insufficient to drive a full blown VTE, or hypercoagulability and require additional cooperating processes (87) A better understanding of such cooperating factors could be crucial for more effective management of VTE in PDAC, or other KRAS driven cancers.

In striving towards better VTE risk stratification in cancer, a comprehensive, large, pan-cancer cohort study (a total of 11,695 patients included) was undertaken to gauge the occurrence of thrombosis as a function of oncogenic mutations (36). The authors explored deep-coverage targeted DNA sequencing data using the Memorial Sloan Kettering-Integrated Mutation Profiling of Actionable Cancer Targets (MSK-IMPACT) platform (36). In their search for somatic mutations associated with VTE risk they revealed some of the previously reported associations, such as KRAS, and MET being correlated with higher VTE risk (62, 88, 89), along with several intriguing new findings (36). For example, they observed that while IDH1 mutations conferred a protective effect against increased VTE risk in glioma patients, as reported earlier (47, 90) this was not the case for other cancers. Overall, this landmark study validated the prediction as to the impact of some (but not all) oncogenic mutations on the VTE risk, but the strength of this association was relatively moderate across different cancer types, again suggesting that additional mechanisms may exist to explain thrombosis in cancer patients.

Not in all cases the mechanistic link between oncogenic mutations and VTE are immediately obvious. On the one hand, studies revealing an impact of IDH1 mutation on VTE suggested that this genetic event may drive methylation-mediated down regulation of two procoagulant effectors, TF and PDPN in cancer cells (6, 60, 91). However, novel links between cancer-associated genetic alterations and VTE risk that have been uncovered by Dunbar et al. (36) often do not point to specific coagulant mechanisms. For example, mutations in STK11, CDKN2B, CTNNB1, KEAP1, and SETD2 are linked to higher risk of VTE across multiple cancers, but their relevant impact on cells and processes involved remains poorly understood. Interestingly, SETD2 mutations, like IDH1 mutations, were found to consistently track with a tendency towards lower VTE risk across a spectrum of malignancies (36). Thus, alternative, possibly non-canonical pro- and anti-coagulant pathways may impinge upon the severity and nature of CAT.

Similarly, thought-provoking are the data related to oncogenic EGFR mutations. While different activated forms of this receptor are prevalent in several cancers (glioma, lung cancer, breast cancer) and have been demonstrated to drive cellular transformation along with elevated TF, factor VII and other elements of the coagulome in cancer cell lines (59), these linkages are more complex in other models and, especially, *in vivo* (6). A part of this discrepancy could lie in the multiplicity of signals received by cancer cells in a complex tissue microenvironment, effects of the epigenome, conventional and EGFR-directed therapies, all of which were previously shown to influence VTE risk (92, 93). Equally puzzling is the JAK2 V617F mutation, which on a large scale bears no association with CAT, but in specific cases of highly prothrombotic states, such as polycythemia vera (PV) is a likely driver of the coagulant phenotype (94, 95). As these correlations become increasingly well-defined and contextualized what remains elusive is the mechanistic basis of VTE in specific cancer settings and this impedes the development of targeted, personalized and more effective CAT management strategies.

### Single cell level heterogeneity of coagulome and unresolved mechanistic questions

Cellular heterogeneity and its evolving nature has only recently been considered in the context of CAT (33). As previously mentioned, tumor microenvironment often exhibits a staggering complexity with a major contribution from host cells to the tumor mass (96). It is therefore highly plausible that when examined as bulk, various subpopulations of cancer cells and tumor-associated stroma, including inflammatory cells contribute, to various extents, to the resulting global tumour coagulome (35).

Single cell sequencing data across a number of cancer studies have repeatedly highlighted the miscellany of cancer cells existing within a particular tumor. For example, the relative proportions and resultant contributions of cellular subpopulations to the global gene expression profile define the nature of GBM subtypes identified thus far (97). In this setting, the emerging model postulates that the phenotypic diversity of cancer cells is to a large degree dictated by both the genome and the epigenome. This is to say that the residual epigenetic differentiation programs, and the ultimate cellular architecture of the tumour mass reflect the biases in the transitory cellular states that are introduced by the oncogenic events driving a particular lesion (98). Thus, the interplay between oncogenic events and epigenetic programs directs the respective tumor cell population towards one subtype or another, albeit without phenotypic uniformity. Moreover, while paracrine effects and stromal cell recruitment are a part of all GBM subtypes, in tumors with mesenchymal signature and frequent loss of NF1 tumor suppressor gene the inflammatory stromal component is especially prominent (35, 98, 99). This exemplifies the impact of the cell-intrinsic tumour cell states on their surrounding host cell compartments, which include the vasculature and the hemostatic system.

Until recently, the implicit consequences for the coagulome, CAT, and VTE risk of this new biology of GBM (and other cancers) emerging out of single cell sequencing and spatial sequencing studies, have rarely been considered (6). However, it is tempting to propose that, the same pressures creating the complex landscape of tumor cells in their various states/phenotypes, would similarly influence the cellular coagulome within GBM, and beyond. In this regard a recent study developed a map of GBM cell population predicated on the differentiation potential of neural stem cells from which these tumours are postulated to originate. This model distinguished several cellular groupings within each lesion, including progenitors (PRO), astrocytic-like cells (AST), oligo-lineage (OLI), neural cells (NEU) and mesenchymal cells (MES). Interestingly, PDPN-positive GBM cells were strongly enriched in MES/MES-like proinflammatory GBM intratumoral cellular compartments (6, 34).

This association at the level of transcriptome suggests that a global coagulome gene expression profile paints only a low-resolution picture of possible CAT related mechanisms involved. If so, it may be worth untangling and discerning what tumor subpopulations are the major contributors to CAT. This, in turn, could make it possible to identify cellular (rather than biochemical) predictors of thrombosis and help develop novel diagnostic tools and interventions compatible with this emerging biology.

While bulk transcriptomics datasets are more readily available and certainly remain of great value, the kind of a global image they project, as mentioned, obscures the cellular complexity of CAT. Therefore, comprehensive single cell-level analysis approaches, such as long-read single cell/nuclei RNA sequencing, single cell DNA sequencing coupled with single cell RNA sequencing, spatial high-resolution sequencing, single cell proteomics, and multi-omics in general may open the access to a completely new highly granular maps of coagulant activities associated with cancer. Such powerful tools would help establish new, and validate the existing, associations between specific oncogenic events and potential drivers of procoagulant phenotype (e.g., involving TF, PDPN, or other effectors). Furthermore, by delving into the actual architecture of tumour tissue such approaches would help overcome limitations of *in vitro* studies and the controlled gene expression approaches, among others. The inevitable reductionist simplicity and artificiality of most *in vitro* (or cell line based) systems often fails to capture the true intricacies and complexities of an actual malignant mass, in contrast to tumor-based single cell profiling approaches which could better discern realistic molecular phenotypes and interactions.

### Cancer-associated thrombosis (coagulome) seen through the lens of single cell sequencing

While DNA single cell sequencing analysis and single cell multi-omics are still at the forefront of development (100, 101), valuable lessons can be drawn from well advanced single cell RNA sequencing techniques. These datasets could provide a glimpse into a deeper nature of correlations between genetic aberrations and CAT in cancer, as well as underlying possible effector mechanisms. To that end, and as proof of principle, we surveyed a number single cell RNAseq datasets of tumors, particularly lung, brain, and pancreatic cancers, in an attempt to capture the cellular landscapes of coagulation-related genes.

**Figure 1:**
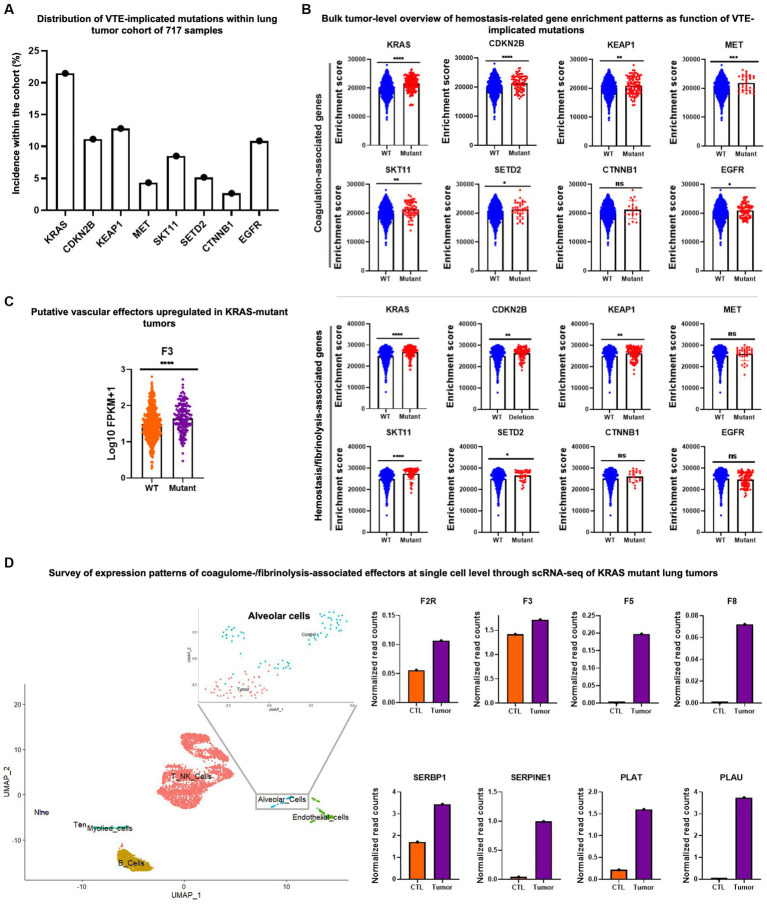
Example of transcriptomic data mining to reveal the single cell coagulome of lung cancer. **(A)** Mutational profile of 717 lung cancer samples. Data was acquired from The Cancer Genome Atlas (TCGA) project and cBioPortal databases. **(B)** Upper panel: single sample gene set enrichment analysis (ssGSEA) of coagulation-associated signature (coagulome) in bulk lung tumor samples segregated by their mutational status (mutant vs. WT) for a given VTE-implicated gene mutation. ssGSEA analysis was conducted using GenePattern (102). Wild type tumors are annotated with blue and mutant tumors are annotated with red. Lower panel: enrichment analysis of fibrinolysis-associated signature (51, 103) using ssGSEA in bulk lung tumor samples segregated by their mutational status (mutant vs. WT) for a given VTE-implicated gene mutation. Wild type tumors are annotated with blue and mutant tumors are annotated with red. **(C)** An example of significantly differentially expressed genes (F3/tissue factor) particularly in KRAS mutant lung cancer compared to KRAS-wild type lung cancer samples. Fragments per kilobase of transcript per million mapped reads (FPKM) values were used to plot gene expression. Orange and purple denote KRAS wild type and mutant tumors, respectively. *T*-test was used to calculate *p*-values where ns, *, **, ***, and **** correspond to *p* > 0.05, *p* ≤ 0.05, *p* ≤ 0.01, *p* ≤ 0.001, *p* ≤ 0.0001, respectively. **(D)** Single cell RNA-seq analysis of KRAS mutant tumors and corresponding matched normal lung tissue (from same patient) obtained from Qian et al. (104) (accession number E-MTAB-6149). Data normalization, clustering, annotations, calculation of average expression of genes in each cell population and identification of differentially expressed genes were done using the Seurat V.3.1.3 bioinformatic tool (105). Uniform manifold approximation and projection (UMAP) visualization was used to show the different cell populations within the dataset including alveolar cell population. A zoom-in on the alveolar cell population clearly shows the segregation of malignant vs. normal alveolar cells. KRAS mutant tumor alveolar cells show a higher average expression level for a number of coagulation and fibrinolysis-associated genes in comparison with the corresponding normal lung alveolar cells.

Thus, to illustrate this point, we re-examined the collection of 717 lung tumor samples with an adequate representation of samples identified as KRAS mutant, as well as samples harboring other oncogenic mutations examined by Dunbar et al. (Figure 1A). We surveyed the published bulk and single cell transcriptome data and we explored the enrichment of gene groups pertinent to coagulation (coagulome), fibrinolysis and other vascular functions in tumor types for which suitable datasets were publicly available (39, 51, 103).

In the context of lung tumors, for example, Dunbar et al. (36) observed the increased VTE hazard ratio in association with KRAS, CDKN2B, KEAP1, MET, and STK11 mutations, and the opposite trend with CTNNB1 and SETD2 mutations. They also pointed at conflicting results in the case of EGFR mutations. Interestingly, coagulation-related genes showed highly significant enrichment in KRAS, CDKN2B, KEAP1, MET and STK11 mutant tumors when compared to their WT counterparts. However, enrichment of moderate to low significance was seen in SETD2 and EGFR mutant tumors and no enrichment in the case of CTNNB1 (Figure 1B, upper panel). This kind of analysis could point to potential procoagulant mechanisms that could be set in motion by various mutational hits and help unveil any previously elusive effectors of coagulant phenotype associated with the respective mutations. These relationships, however, do not reveal in which cells the relevant genes were mutated or expressed, crucial information that should be validated at the single cell level.

Similar to coagulation-related gene enrichment, the expression of fibrinolysis- and hemostasis-associated genes was significantly linked to KRAS, CDKN2B, KEAP1, and STK11 mutations, but not to MET mutation. In SETD2, CTNNB1 and EGFR mutant tumors, fibrinolysis-related gene enrichment was of low/no significance (Figure 1B, lower panel). Among coagulation-related genes, *F3*/*TF* is highly significantly over-expressed in KRAS mutant tumors (F3/TF being directly involved in coagulation) when compared to tumors identified as WT for KRAS (Figure 1C).

To highlight the importance of the cellular heterogeneity aspect of cancer coagulome we re-examined lung cancer single cell RNA seq data containing cells extracted from KRAS mutant tumors and corresponding normal lung tissues from each patient (104). We observed that among the cells isolated from the lung tumor masses, particularly the subpopulation identified as alveolar cells, showed considerably higher average expression of coagulation- and hemostasis/vascular regulation-related genes. Multiple effectors showed a trend of upregulated expression in tumor alveolar cell subsets in comparison to normal alveolar cells (Figure 1D).

While all cells considered in this aforementioned analysis came from KRAS mutant tumors, their individual mutational status is often difficult to ascertain. This is because the 3′ and 5′ short reads generated by most commonly employed 10X chromium sequencing platform along with limited depth of current single cell RNA sequencing approaches result in a very weak capacity to track mutations, variants, splicing and other structural alterations (106), which prohibits the validation of a given cell as being positive or negative for KRAS mutation. This is where, as alluded to earlier, it would be of great utility to be able to use the emerging single cell multi-omics and in-tandem DNA and RNA single cell analysis approaches.

In the frame of brain tumors, *CDKN2B* and/or *CDKN2A* mutations are among the most frequent events seen in 50%–60% of all GBMs (107). In this case, homozygous deletions are most frequently observed in GBM subtypes originally denoted as Mesenchymal and RTKI-II (108) (currently assigned to MES and CL subgroups, respectively (54)). The assignment of these mutations and their coagulation-related consequences to single cell datasets and to emerging models of GBM requires some consideration of historical complexities surrounding GBM classification. Thus, while RTK-type GBM annotations were based on methylome-based studies, the RTKI subtype of GBM has been shown to coincide with a cluster of tumors with PDGFRA amplification (108) akin to proneural subtype, as defined by the initial TCGA classification (109). This assignment is confusing, as it initially encompassed IDH1/2 mutant HGG [now excluded from GBM diagnosis (54)] and known to be non-thrombotic. Nonetheless, the designation of a subset of tumors as proneural meant to signal that these tumors resemble the gene expression profiles of a subset of cells that were believed to be enriched in these cancers and were characterized as oligodendrocyte progenitor-like cells (OPC-like). Indeed, OPCs are present in GBM, but constitute a major or minor subpopulation depending on underlying mutational profile as recently proposed by Neftel et al. (98, 109). In contrast to proneural GBMs (enriched in OPCs) the RTKII GBM subtype, is enriched for cells with astrocytic (AC-like) transcriptional program and associated with a preponderance of EGFR amplification (98, 109) and often deleted for *CDKN2B* (110). Interestingly, Dunbar et al. identified a trend toward an increase in VTE hazard ratio with the presence of *CDKN2B* mutations in GBM, the majority of which present as deletions.

**Figure 2:**
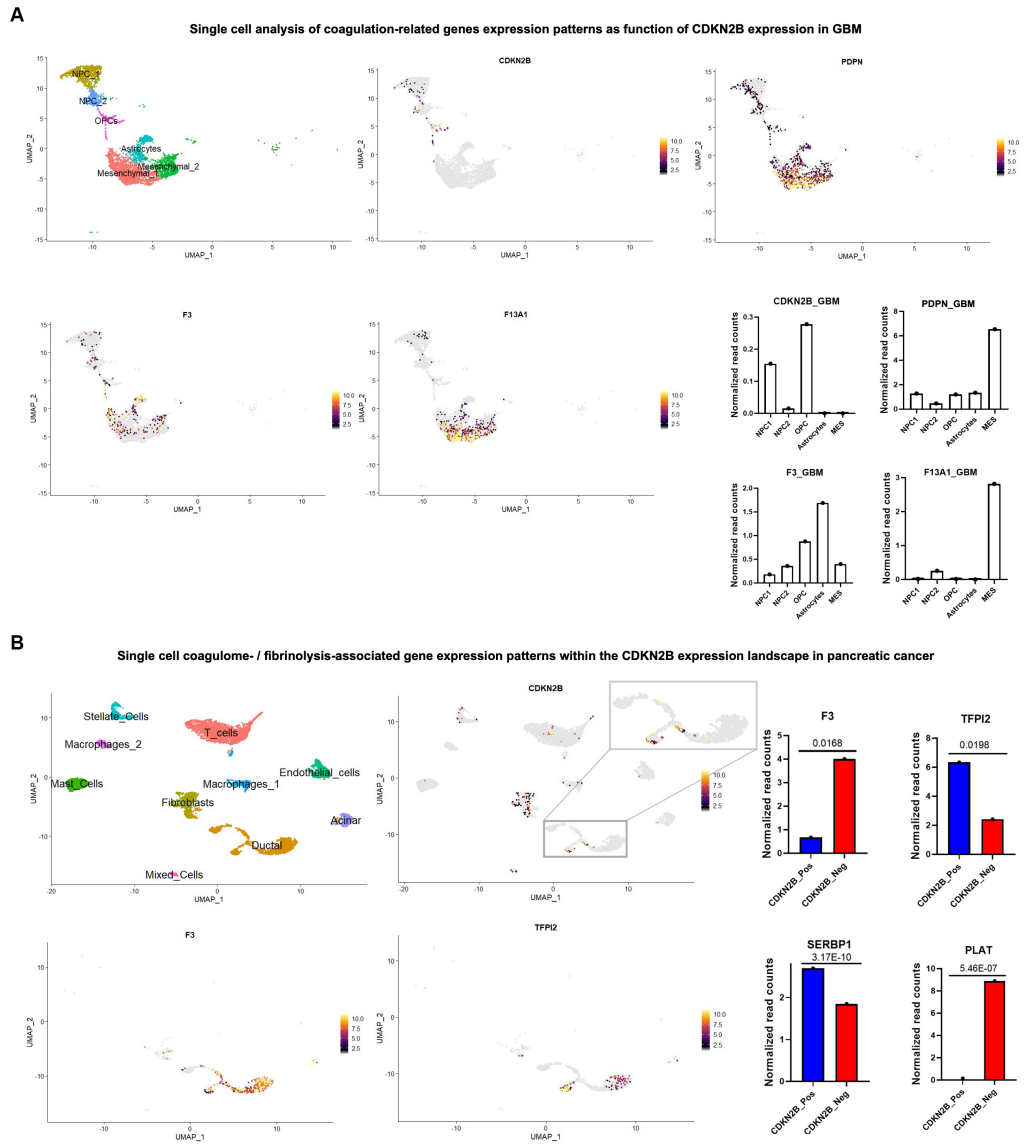
Single cell sequencing reveals complex coagulant phenotypes of cancer cell subpopulations driven by loss of *CDKN2B* tumour suppressor gene. Single cell/nuclei RNA-seq analysis was used and in both GBM and pancreatic cancer datasets; data normalization, clustering, annotations and calculation of average expressions and marker genes were done using Seurat V.3.1.3 (105). **(A)** UMAP visualization of single nuclei RNA-seq of GBM dataset from Neftel et al. (98) (accession number GSE131928) shows the heterogenous tumor composition and presence of different cell populations including, NPCs, OPCs, astrocytes and mesenchymal cell subsets. Expression patterns of *CDKN2B* and three prominent coagulation effectors (PDPN, F3/tissue factor and F13A1, the latter a catalyst of fibrin cross linking) were surveyed across different cell populations and the corresponding normalized read counts are shown in adjacent graphs. **(B)** Pancreatic cancers (accession number: GSE214295) were analyzed and the various cell populations constituting the tumors were annotated in the UMAP. Among *bona fide* tumor cells, a zoom-in on the tumor cell subset identified as ductal cells showed a relatively higher presence of CDKN2B positive cells. CDKN2B positive and negative cells were compared, and examples of differentially expressed hemostasis-related genes were pinpointed, including F3/TF, PLAT, TFPI2, and SERBP1. *p*-values were calculated using FindMarkers function in Seurat package.

Based on these considerations we revisited the GBM single cell sequencing data set published by Neftel et al. (109, 111), mapped *CDKN2B* expression, and surveyed the expression of coagulation related genes. *CDKN2B* deletions have been previously identified to track most tightly with the mesenchymal and classical/astrocytic subtypes of GBM, and indeed, at the single cell level, loss of expression was found to dominate the astrocytic and mesenchymal cell clusters, while any remaining expression was limited to neural progenitor cells (NPC) and, to a lesser extent, to OPC clusters of cells (Figure 2A). The expression of coagulation-associated genes on the other hand, followed the opposite trend where the astrocytic and mesenchymal clusters that showed lack of *CDKN2B* expression exhibited a significant concentration of cells with high procoagulant gene expression, including, but not limited to PDPN, which has been proposed as potential major contributor to CAT in GBM (Figure 2A) (6, 57).

Remarkably, and in a trend opposite to what was seen in brain tumors, Dunbar et al. (36) found the presence *CDKN2B* loss of function mutations in pancreatic cancer to be of a slightly protective nature against VTE. However, looking at a single cell RNA seq data set of pancreatic cancers (GSE214295), the absence of *CDKN2B* expression was prominently observed in the cell population identified as ductal cells, with a more complex expression pattern of coagulation related genes. For example, *F3* (TF)—the initiator of the coagulation cascade—was highly overexpressed in *CDKN2B* negative ductal cells, which also expressed transcripts for the tissue type plasminogen activator (PLAT)—a catalyst of plasmin generation, which drives clot breakdown. Other hemostasis regulators, such as SERBP1 and TFPI2, exhibited a diminished levels in the absence of *CDKN2B* expression (Figure 2B). Thus, specific molecular events can drive antithetical gene expression responses, both procoagulant and fibrinolytic in the same cancer cells. These opposing coagulant functionalities and their regulatory feedback loops may cumulatively contribute to dysregulation of hemostasis and exceedingly high VTE risk (perhaps through a unique cellular mechanism) in this disease (11). These explorations exemplify how single cell analysis may offer unparalleled insights into how oncogenic events intersect with cellular and multicellular processes regulating hemostatic responses in different cancers.

## Conclusion

In this article we offer a new glimpse into the cellular complexity and potential diversity of cellular mechanisms involved in driving CAT in various cancer settings. In the recent landmark study by Dunbar and colleagues, deep-coverage targeted DNA sequencing (MSK-IMPACT) was used to identify mutational status of various cancer cases and link them to the overall VTE risk. However, we would suggest that bulk DNA/RNA sequencing, or even the emerging single cell RNA sequencing datasets may not be sufficient to understand causal influences cancer cells exert upon the pathogenesis of CAT. In bulk approaches, cellular details of coagulant gene expression are obscured, such that dominantly procoagulant, CAT-driving cellular populations cannot be reliably or directly identified. Consequently, solid correlations, especially as to mechanistic causation may be difficult, or impossible to draw, and small populations of highly procoagulant cells may be missed. Standard single cell RNA sequencing on the other hand, when considered alone, seldom allows for correct mutational status identification, and hence any correlations of procoagulant gene expression profiles with genetic aberrations could be compromised by analytical factors. Moreover, the correlative nature of such studies impedes a direct validation of the link between the repertoire of candidate genes and their actual functionality.

**Figure 3:**
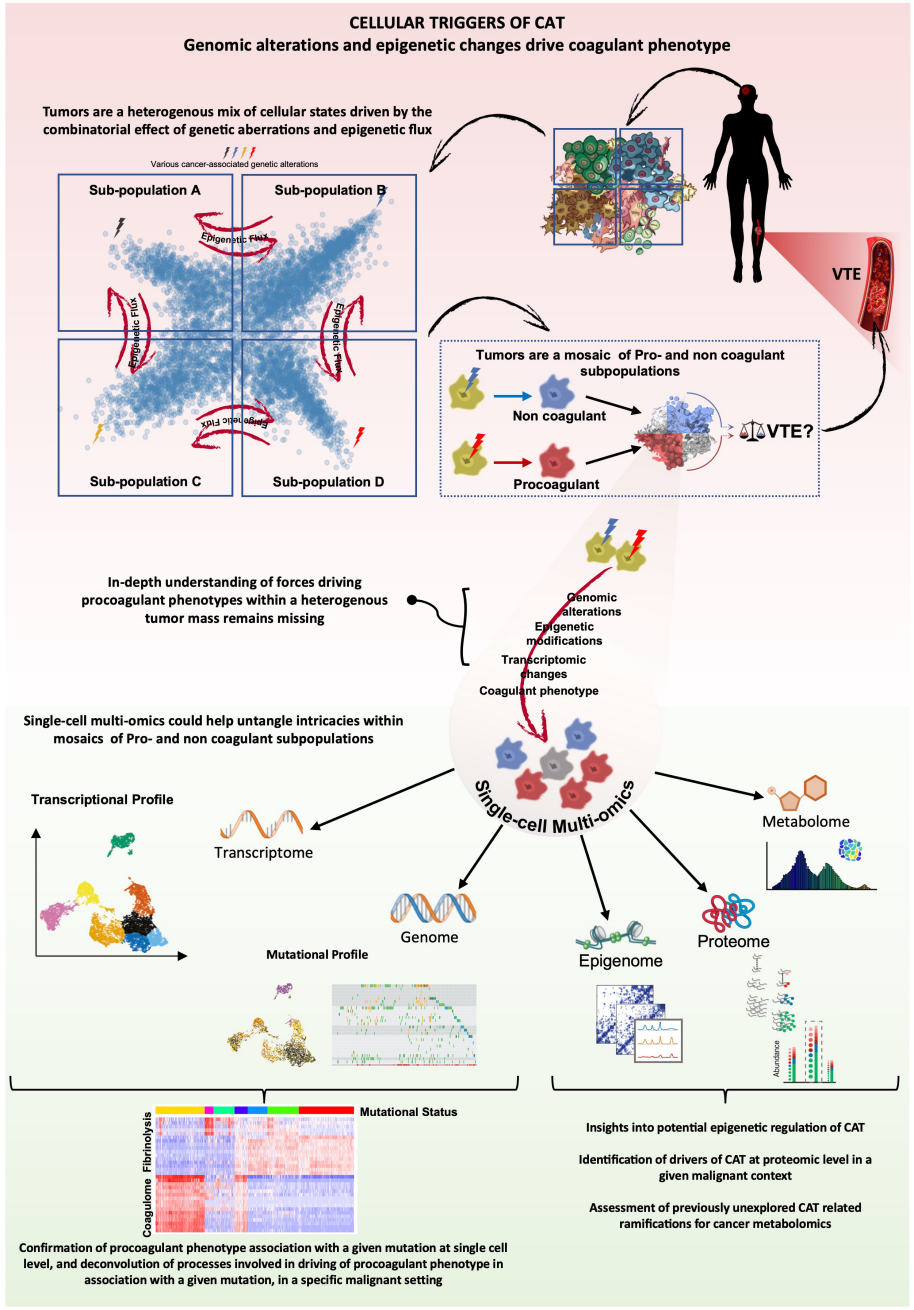
CAT analysis through the lens of cancer multiomics cancer-associated genomic alterations have been correlated with VTE risk, however progress towards elucidating direct cause-effect relationships and underlying mechanisms remains hindered by tumor complexity and the relative scarcity of datasets combining genomic and transcriptomic data. Cancer heterogeneity extends to coagulant phenotype, and accordingly, tumors are often composites of procoagulant and non-coagulant tumor subpopulations or mixed phenotypes. The cumulative combinatorial effect of the heterogenous expression of the respective coagulant phenotypes within a tumor could thus potentially dictate the extent and nature of CAT, impacting VTE risk. Single-cell multi-omics, particularly the integration of single-cell genomics and transcriptomics, offers a unique opportunity to extract cause-effect relationships between various genetic alterations and coagulant phenotypes of cells and reveal putative, context-specific, molecular drivers of CAT across a spectrum of malignant diseases. Other elements of single-cell multi-omics could further enhance our understanding of epigenetic regulation of potential effectors of CAT and possibly generate insights towards previously unexplored areas like the impact a procoagulant tumor subpopulation could have on the metabolomic landscape within the tumor and disease progression in general.

We would like to postulate that with some refinements, genomic approaches could, however, redefine our understanding of the nature of CAT and its manifestations, such as hypercoagulable state and VTE. A combined DNA and RNA single cell analysis and multi-omics, in general, as well as long read single cell RNA sequencing technologies that are beginning to emerge and increasingly enter clinical practice (106), could pave the way towards improved molecular predictions and more validation and/or discovery of real relationships underlying thrombosis. Thus, a more accurate analysis of correlations between molecular landscapes and VTE risks in specific cancers, along with identification of procoagulant cellular subpopulations (be it cancer or host-derived), could lead to a re-definition of procoagulant phenotypes within the frame of specific malignancies. In addition, spatial single cell transcriptomics and proteomics may reveal regional distribution of CAT-driving cellular populations and their interactions with the vascular system. These read outs could be eventually interlinked with proteomic and targeted screens of plasma to develop tools for both VTE prediction and molecular causation in CAT (112) (Figure 3). Within this framework, once robust correlations are established and validated, liquid biopsy analytes—such as circulating DNA, RNA and extracellular vesicles—which often provide insight into the transformed state of cancer cells, hold the potential to serve not only as distinctive biological ‘fingerprints’ for cancer itself but also as putative indicators of CAT risks.

Finally, the possible cooperation or antagonisms between different cell subsets, or within them, could also be computed from such multidimensional datasets (6). With new technologies entering the field and sequencing platforms making inroads within the clinical practice landscape, it may be possible to peer more deeply into the nature of CAT in individual patients, improve VTE risk assessment, personalize care and thereby off-set the impact of thrombosis on cancer mortality and morbidity.

## Author contributions

All authors listed have made a substantial, direct, and intellectual contribution to the work and approved it for publication.
